# LAPAROSCOPIC VERSUS OPEN SURGERY IN GASTRIC GASTROINTESTINAL STROMAL TUMORS LARGER THAN 5 CM: A SYSTEMATIC REVIEW AND META-ANALYSIS

**DOI:** 10.1590/0102-672020220002e1711

**Published:** 2023-01-09

**Authors:** Francisco Antonio PITA ARAUJO, Vítor Nuno Neves LOPES, Jose Pedro Coimbra de Vargas Lobarinhas BARBOSA, Mariana Rafaela da Fonte MARTINS, José BARBOSA

**Affiliations:** 1 Universidade do Porto, Faculty of Medicine – Porto, Portugal;; 2 Universidade do Porto, Faculty of Medicine, Department of Surgery and Physiology – Porto, Portugal;; 3 Department of General Surgery, São João University Hospital Center – Porto, Portugal;; 4 Universidade do Porto, Faculty of Medicine, São João University Medical Center, Department of Community Medicine, Information and Decision in Health – Porto, Portugal.

**Keywords:** Gastrointestinal Stromal Tumors, Laparotomy, Gastrectomy, Minimally Invasive Surgical Procedures, Review, Tumores do Estroma Gastrointestinal, Laparotomia, Gastrectomia, Procedimentos Cirúrgicos Minimamente Invasivos, Revisão

## Abstract

**BACKGROUND:**

Surgical resection represents the main treatment for resectable nonmetastatic gastric gastrointestinal stromal tumors. Despite the feasibility and safety of laparoscopic resection, its standard use in gastric tumors larger than 5 cm is yet to be established.

**AIMS:**

This study aimed to compare the current evidence on laparoscopic resection with the classical open surgical approach in terms of perioperative, postoperative, and oncological outcomes.

**METHODS:**

The PubMed, Scopus, and Web of Science databases were consulted. Articles comparing the approach to gastric gastric gastrointestinal stromal tumors larger than 5 cm by open and laparoscopic surgery were eligible. A post hoc subgroup analysis based on the extent of the surgery was performed to evaluate the operative time, blood loss, and length of hospital stay.

**RESULTS:**

A total of nine studies met the eligibility criteria. In the study, 246 patients undergoing laparoscopic surgery and 301 patients undergoing open surgery were included. The laparoscopic approach had statistically significant lower intraoperative blood loss (p=0.01) and time to oral intake (p<0.01), time to first flatus (p<0.01), and length of hospital stay (0.01), compared to the open surgery approach. No significant differences were found when operative time (0.25), postoperative complications (0.08), R0 resection (0.76), and recurrence rate (0.09) were evaluated. The comparative subgroup analysis between studies could not explain the substantial heterogeneity obtained in the respective outcomes.

**CONCLUSION:**

The laparoscopic approach in gastric gastrointestinal stromal tumors larger than 5 cm compared to the open surgical approach is a technically safe and feasible surgical method with similar oncological results.

## RESUMO

**RACIONAL:**

A resseção cirúrgica representa a principal forma de tratamento de tumores estromais gástricos não metastáticos ressecáveis. Apesar da viabilidade e segurança da ressecção laparoscópica, a sua utilização generalizada em tumores gástricos com mais de 5 centímetros ainda não foi estabelecida.

**OBJETIVOS:**

Comparar as evidências atuais sobre a resseção por via laparoscópica com a cirurgia aberta, em termos de resultados peri-, pós-operatórios e oncológicos.

**MÉTODOS:**

Foram consultadas as bases de dados PubMed, Scopus e Web of Science. Artigos que comparassem a abordagem de tumores estromais gástricos gástricos com tamanho superior a 5 centímetros por cirurgia aberta e por via laparoscópica foram incluídos. Uma análise de subgrupos *post-hoc,* com base na extensão da cirurgia, foi realizada para os outcomes tempo intra-operatório, perdas de sangue e tempo de hospitalização.

**RESULTADOS:**

Nove estudos cumpriram os critérios de elegibilidade, tendo sido incluídos 246 pacientes submetidos a cirurgia por via laparoscópica e 301 pacientes submetidos a cirurgia aberta. A abordagem laparoscópica apresentou perdas de sangue intra-operatórias (p=0,01) e tempos até alimentação oral (p<0,01), para primeiro flato (p<0,01) e de hospitalização (0,01) estatisticamente inferiores relativamente à abordagem por cirurgia aberta. Não foram encontradas diferenças significativas quando avaliados o tempo operatório (0,25), complicações pós-operatórias (0,08), resseção R0 (0,76) e taxa de recorrência (0,09). A análise de subgrupos comparativa não permitiu explicar a heterogeneidade substancial obtida nos respetivos *outcomes*.

**CONCLUSÕES:**

A via laparoscópica em tumores estromais gástricos superiores a 5 centímetros comparativamente com a abordagem por cirurgia aberta, constitui um método cirúrgico tecnicamente seguro e viável, com resultados oncológicos semelhantes.

## INTRODUCTION

Gastrointestinal stromal tumors (GISTs), which originate from the interstitial cells of Cajal, located in its muscular layer, are the most frequent malignant subepithelial lesions (SELs) of the gastrointestinal (GI) tract. They are characterized by overexpression of the tyrosine kinase receptor KIT and, although they can arise in any area of the GI tract, most are found in the stomach (60%), followed by the small intestine (30%), colon (7%), rectum (5%), and esophagus (1%)^1,21^.

Despite tyrosine kinase inhibitors such as imatinib are currently the treatment of choice for metastatic or recurrent GISTs, surgical resection is still considered the first choice in cases concerning nonmetastatic resectable tumors. The goal of the surgery is to achieve complete resection with free margins, and lymphadenectomy is usually not necessary^1,6,26,29^.

When, initially, characterization of a GIST is the intention, it is considered that simply labeling the tumor as benign or malignant may not be the most appropriate approach, as even small tumors with low mitotic counts can sometimes metastasize and have malignant potential. Therefore, GISTs risk stratification (very low, low, intermediate, or high) seems to be more appropriate, with the variables considered as predictors of aggressive clinical behavior being tumor size 5 cm or larger and a mitotic index of at least 5 mitoses/50 HPF (high-power field)^10,14,15,26^.

With the progress of minimally invasive surgical approaches, laparoscopic surgery (LAP) for small-sized gastric GISTs has proven to be a viable and safe option with oncological outcomes comparable to traditional open surgery (OS). However, while at first it was thought that 2 cm was the upper limit for resection by laparoscopic approach, being its choice for large tumors even discouraged^28^, this size limit has been put into question, with several authors demonstrating that laparoscopic resection of tumors larger than 5 cm can be an option^3,5,24^.

The aim of this systematic review and meta-analysis was to compare the current evidence on laparoscopic resection with the classical open surgical approach, in terms of perioperative and oncological outcomes, seeking to confirm its feasibility and safety in gastric GISTs larger than 5 cm.

## METHODS

This systematic review was based on the Preferred Reporting Items for Systematic Reviews and Meta-Analyses (PRISMA) guidelines^19^.

### Eligibility Criteria of Primary Studies

As eligible articles for this review, we considered randomized controlled trials (RCTs) and observational nonrandomized clinical trials, which compared the laparoscopic (intervention) and open surgical (comparator) approaches to histologically confirm gastric GISTs larger than 5 cm (population). Only articles in which it was possible to access the full text were included. Studies that (1) were related to metastatic cancer, (2) did not present any of the outcomes being evaluated, and (3) compared different techniques (e.g., endoscopic route) were excluded.

### Search Strategy

Studies were identified by searching PubMed, Web of Science, and Scopus, with the recent survey conducted on January 29, 2022. Regarding the search strategy used in PubMed, it was as follows: (“stomach”[MeSH Terms] OR “stomach”[Text Word] OR “gastric*”[Text Word]) AND (“Gastrointestinal Stromal Tumors”[MeSH Terms] OR “gastrointestinal stromal tumor*”[Text Word] OR “GISTs”[Text Word] OR “GIST”[Text Word]) AND (“gastrectomy”[MeSH Terms] OR “laparotomy”[MeSH Terms] OR “laparotom*”[Text Word] OR “open surger*”[Text Word] OR “open resection*”[Text Word] OR “tumor resection*”[Text Word]) AND (“minimally invasive surgical procedures”[MeSH Terms] OR “laparoscopy”[MeSH Terms] OR “laparoscopic surger*”[Text Word] OR “laparoscopic surgical procedure*”[Text Word]). The search strategy used in Scopus was as follows: [ALL (gastric OR stomach) AND ALL (gist OR gastrointestinal stromal tumors) AND ALL (open surgery OR laparotomy OR tumor resection) AND ALL (laparoscopic surgery OR laparoscopy OR minimally invasive surgical procedures)]. In Web of Science, the search strategy was as follows: (TS=(stomach) OR TS=(gastric)) AND (TS=(Gastrointestinal Stromal Tumor) OR TS=(GIST)) AND (TS=(laparotomy) OR TS=(open surgery) OR TS=(Tumor resection) OR TS=(Open resection)) AND (TS=(Laparoscopic surgery) OR TS=(laparoscopy) OR TS=(minimally invasive surgical procedures)).

Studies published until January 2022 were included. No language restrictions were applied. Complementarily, during writing, some works cited in the selected articles were consulted.

### Study Selection and Data Extraction Process

After exclusion of duplicates, the initial screening and interpretation process of the studies were done based on their titles and abstracts by two independent reviewers. Disagreements were resolved by consensus after discussion among reviewers. Subsequently, the selected articles were read in their entirety. This phase was also carried out by two independent reviewers.

The clinical outcomes assessed are as follows:

1. intraoperative outcomes (operative time and intraoperative blood loss);
2. short-term postoperative outcomes (time to oral intake, time to first flatus, and length of hospital stay);
3. postoperative complications; and
4. oncological outcomes (R0 resection and recurrence rate).

Data extraction was performed independently by two reviewers. We contacted another author, via email, for further information, but he was not able to provide the requested information. Other data were extracted in addition to the outcomes being evaluated, including basic study information (author, study design type, study period, geographic region, follow-up, sample size of each intervention) and population characteristics (patient age, gender, mitotic rate, and tumor size).

### Statistical Analysis and Quality Assessment

To perform the data analysis, the Review Manager (RevMan) (Computer program, version 5.4) software was adopted. The 2020 Cochrane Collaboration was used, and the meta-analysis was developed based on the format described in the Handbook^7^ made available by the “The Cochrane Collaboration”. Mean difference (MD) was calculated as a measure of effect for the analysis of continuous variables (operative time, intraoperative blood loss, time to oral intake, time to first flatus, and length of hospital stay), and risk ratio (RR) was used for dichotomous variables (postoperative complications, R0 resection, and recurrence rate). Hozo et al.^11^ described a method that allows estimation of the mean and standard deviation from median and range values, and this was applied in our review in studies that did not report these measures of effect. Statistical significance was defined as p<0.05 and the confidence interval (CI) was set at 95%. Cochran’s Q test and I^2^ were used to evaluate the heterogeneity of the studies. We considered substantial heterogeneity when I^2^>50% (or p<0.10 in the Q test). In these cases, the random-effects model was used. In the absence of substantial heterogeneity (I^2^=50% or p>0.10 in the Q test), the fixed-effects model was applied. Finally, to explore high levels of heterogeneity, a post hoc subgroup analysis was performed for the outcomes with substantial heterogeneity (I^2^>50%). The studies were grouped into two subgroups:

1. studies that only reported results regarding atypical gastrectomies (wedge resection); and
2. studies that included all types of gastrectomies (total, proximal subtotal, distal subtotal, and atypical).

After the literature search, no RCTs were identified that fit the criteria of this systematic review, so only observational studies were used. To proceed with the quality assessment of these studies, the Methodological Index for Non-Randomized Studies (MINORS) checklist^25^, which is based on 12 items, was used by two independent reviewers. Each study can obtain a total score of 24, and for each item described in the checklist, a score of 0 (not reported), 1 (reported but inadequate), or 2 (reported and adequate) is assigned.

## RESULTS

### Search Results

**Figure 1.**
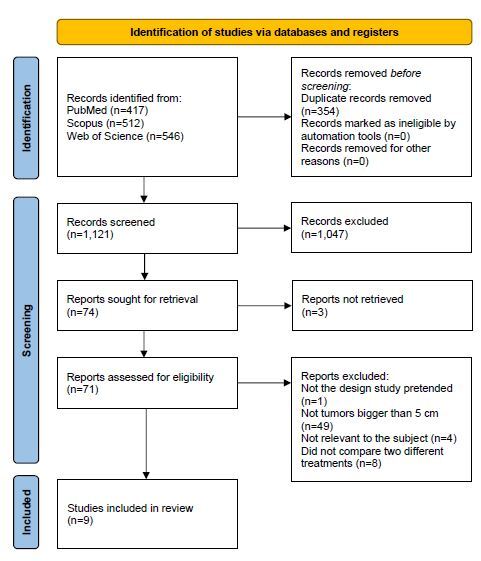
Flowchart according to the PRISMA guidelines.

The initial search of the PubMed, Web of Science, and Scopus platforms revealed 417, 546, and 512 studies, respectively, for a total of 1475 potentially relevant articles. Of the total, 354 were excluded as duplicates. A total of 1047 articles were excluded after reading the title/abstract, and from 71 full-text articles analyzed, a total of 9 studies^12,16-18,20,22,23,27,28^ were obtained that met the eligibility criteria for the qualitative and quantitative analysis. The results of the studies were mostly published in English, with only one being in Chinese. Figure 1 shows the flowchart explaining the reasons that, at each step of the process, led to the exclusion of the remaining articles.

PRISMA: Preferred Reporting Items for Systematic Reviews and Meta-Analyses.

### Characteristics of the Included Studies

All studies were published between 2012 and 2017. Nine retrospective cohort studies^12,16-18,20,22,23,27,28^ (four from China, one from France, one from Japan, one from Taiwan, one from Korea, and one from Singapore) were used to perform the meta-analysis. Sample sizes ranged from 26 to 183, involving a total of 246 patients undergoing LAP and 301 patients undergoing OS, and data were extracted from a total of 547 patients. After surgical intervention, median follow-up ranged from 20.5 to 78 months. The characteristics of each study are summarized in Table 1.

**Table 1.** Included studies in the systematic review and respective characteristics.

Study (author)	Year	Study design	Country	Years of enrollment	Intervention (n)	Comparison (n)	Outcomes	Sample size, n	Median follow-up (months) (range)	
									LAP	OS
Kim et al.	2012	ROS	Korea	1998–2011	*LAP* (n=24)	*OS* (n=14)	*A, E, F, G*	48	62.6(8.9–164.4)	58.3(18.8–123.2)
Hsiao et al.	2015	ROS	Taiwan	2002–2012	*LAP* (n=18)	*OS* (n=21)	*A, B, F, G*	39	37.2(16.8–133.2)	67.2(12–133.2)
Lin et al.	2014	ROS	China	2007–2012	*LAP* (n=23)	*OS* (n=23)	*A, B, C, D, E, F, G*	46	34(6–78)	34(6–78)
Takahashi et al.	2015	ROS	Japan	1995–2011	*LAP* (n=12)	*OS* (n=15)	*A, B, E, F, G*	27	57(7–120)	69(13–154)
Piessen et al.	2015	ROS	France	2001–2013	*LAP* (n=90)	*OS* (n=93)	*E, G*	183	NR	NR
Xue et al.	2015	ROS	China	2008–2013	*LAP* (n=19)	*OS* (n=62)	*A, B, D, F,*	81	25(7–64)	47(7–84)
Khoo et al.	2017	ROS	Singapore	2002–2015	*LAP* (n=23)	*OS* (n=36)	*A, B, C, E, F, G*	59	20.5(0–163)	78(2–151)
Qiu et al.	2017	ROS	China	2008–2014	*LAP* (n=24)	*OS* (n=24)	*A, B, C, D, E, F, G*	48	50	52
Lian et al.	2017	ROS	China	2008–2015	*LAP* (n=13)	*OS* (n=13)	*A, B, C, D, E, F, G*	26	48(26–78)	42(11–83)

ROS: retrospective observational study; LAP: laparoscopic surgery; OS: open surgery; NR: not reported; A: operative time, B: intraoperative blood loss, C: postoperative time to oral intake, D: postoperative time to first flatus, E: postoperative complications, F: postoperative hospital stay; G: recurrence rate.

Considering that we do not have the data regarding the patients’ age and gender for two studies (Piessen et al.^22^ and Xue et al.^28^), a total of 152 female patients (77 from LAP; 75 from OS) and 131 male patients (60 from LAP; 71 from OS) participated in the remaining studies, with the mean and median age ranging from 50 to 70 years. All studies reported data for tumors larger than 5 cm. The baseline characteristics of the patients included are summarized in Table 2. Table 3 shows the postoperative complications rates, recurrence rates, and R0 resection rates and Table 4 lists the mean and standard deviations of operative time, intraoperative blood loss, time to oral intake, time to first flatus, and length of hospital stay. Regarding the analysis of the methodological quality of the studies, all of them scored 17 or higher on the MINORS checklist, thus ensuring the high quality of all studies that have been considered into our review (Table 5).

**Table 2.** Summary of baseline characteristics.

Study (author)	Age (mean±SD)		Sex (M/F)		Tumor size (cm) (mean±SD)		Mitotic rate			
	LAP	OS	LAP	OS	LAP	OS	LAP		OS	
							<5/ 50 HFPs	>5/ 50 HFPs	<5/ 50 HFPs	>5/ 50 HFPs
Kim et al.	57.4±8.1	65.9±12.2	12/12	4/10	6.1±1.3	7.2±1.7	16	8	5	9
Hsiao et al.	66.6±14	64.5±10.4	8/10	7/14	6.3±1.1	6±0.9	14	4	17	4
Lin et al.	63.4±12.9	62±11.3	11/12	16/7	7.2±1.6	7.3±1.5	16	7	13	10
Takahashi et al.	64 (18-78)*	66 (37–76)*	7/5	10/5	5.5 (5.1–5.7)*	7.5 (5.3–13)*	9	3	10	5
Piessen et al.	NR	NR	NR	NR	NR	NR	NR	NR	NR	RN
Xue et al.	NR	NR	NR	NR	NR	NR	NR	NR	NR	NR
Khoo et al.	61 (31–86)*	66.5 (21–90)*	9/14	19/17	6 (5–11)*	6.25 (5–12.5)*	10	10	13	21
Qiu et al.	65.9±7.9	61.5±10.9	8/16	11/13	7±1.4	7.6±1.75	19	5	16	8
Lian et al.	56.15±12.84	60.73±9.32	5/8	4/9	6 (5–11)	6 (5–11)	6	7	5	8

HFPs: high-power fields; LAP: laparoscopic surgery; OS: open surgery; M: male; F: female; SD: standard deviation; NR: not reported. *Data are represented as median (range).

**Table 3.** Summary data for dichotomous outcomes: (A) postoperative complications rates; (B) recurrence rates; and (C) R0 resection rates.

Study	Laparoscopic surgery n (%)	Open surgery n (%)
(A)		
Kim et al., 2012	1/24 (4.2%)	0/14 (0.0%)
Lin et al., 2014	2/23 (8.7%)	3/23 (13.0%)
Takahashi et al., 2015	1/12 (8.3%)	1/15 (6.7%)
Piessen et al., 2015	11/90 (12.2%)	21/93 (22.6%)
Khoo et al., 2017	2/23 (8.7%)	5/36 (13.9%)
Qiu et al., 2017	3/24 (12.5%)	5/24 (20.9%)
Lian et al., 2017	1/13 (7.7%)	0/13 (0.0%)
(B)		
Kim et al., 2012	1/24 (4.2%)	4/14 (28.6%)
Lin et al., 2014	3/23 (13.0%)	5/23 (21.7%)
Hsiao et al., 2015	1/18 (5.6%)	0/21 (0.0%)
Takahashi et al., 2015	1/12 (8.3%)	2/15 (13.3%)
Piessen et al., 2015	5/90 (5.6%)	7/93 (7.5%)
Khoo et al., 2017	0/23 (0.0%)	2/36 (5.6%)
Qiu et al., 2017	1/24 (4.2%)	2/24 (8.3%)
Lian et al., 2017	0/13 (0.0%)	1/13 (7.7%)
(C)		
Kim et al., 2012	24/24 (100%)	14/14 (100%)
Hsiao et al., 2015	17/18 (94.4%)	19/21 (90.5%)
Qiu et al., 2017	23/24 (95.8%)	23/24 (95.8%)
Lian et al., 2017	13/13 (100%)	13/13 (100%)

**Table 4.** Summary data for continuous outcomes: (A) operative time; (B) intraoperative blood loss; (C) time to oral intake; (D) time to first flatus; and (E) length of hospital stay.

(A)						
Study	Laparoscopic surgery		Open surgery			
	Mean(min)	SD(min)	Sample(size)	Mean(min)	SD(min)	Sample(size)
Kim et al., 2012	119.5	62.2	24	154.3	53.5	14
Lin et al., 2014	124.1	50.3	23	196.5	64.8	23
Hsiao et al., 2015	146.6	50.2	18	113.3	42.9	21
Takahashi et al., 2015	123.75*	44.75*	12	119.5*	44.22*	15
Xue et al., 2015	128.9	38.2	19	106	39.2	62
Khoo et al., 2017	210*	81.25*	23	105*	48.75*	36
Qiu et al., 2017	131	44	24	103	30	24
Lian et al., 2017	197.46	59.774	13	129.23	56	13

(B)						
Study	Laparoscopic surgery		Open surgery			
	Mean(mL)	SD(mL)	Sample(size)	Mean(mL)	SD(mL)	Sample(size)
Lin et al., 2014	35.6	28.3	23	127.8	116.8	23
Hsiao et al., 2015	42.2	47.7	18	51.4	58.4	21
Takahashi et al., 2015	202.5*	244.65*	12	255*	262.69*	15
Xue et al., 2015	34.1	26.5	19	60.4	60.4	62
Khoo et al., 2017	78.26*	75*	23	128.47*	125*	36
Qiu et al., 2017	73	36	24	105	51	24
**Lian et al., 2017**	**207.5**	**175.86**	**13**	**105**	**86.84**	**13**

(C)						
Study	Laparoscopic surgery		Open surgery			
	Mean(days)	SD(days)	Sample(size)	Mean(days)	SD(days)	Sample(size)
Lin et al., 2014	2.3	1.5	23	3.5	2.3	23
Khoo et al., 2017	2.25*	0.75*	23	3*	1.25*	36
Qiu et al., 2017	3.2	0.6	24	3.6	0.8	24
Lian et al., 2017	4.77	1.48	13	4.23	2.45	13

(D)						
Study	Laparoscopic surgery		Open surgery			
	Mean(days)	SD(days)	Sample(size)	Mean(days)	SD(days)	Sample(size)
Lin et al., 2014	1.9	1.2	23	3	1	23
Xue et al., 2015	3.5	1.4	19	3.9	1	62
Qiu et al., 2017	2.2	0.5	24	2.5	0.7	24
Lian et al., 2017	3.69	0.75	13	3.92	1.66	13

(E)						
Study	Laparoscopic surgery		Open surgery			
	Mean(days)	SD(days)	Sample(size)	Mean(days)	SD(days)	Sample(size)
Kim et al., 2012	4.8	1.8	24	9.2	3.2	14
Lin et al., 2014	124.1	50.3	23	196.5	64.8	23
Hsiao et al., 2015	8.4	2.9	18	9.6	2.4	21
Takahashi et al., 2015	14.25*	7.46*	12	18.25*	8.01*	15
Xue et al., 2015	7.2	3.8	19	9	4.8	62
Khoo et al., 2017	221.22*	17.5*	23	21.12*	15*	36
Qiu et al., 2017	6.6	2.2	24	8.1	2.3	24
Lian et al., 2017	7.92	2.66	13	6.69	1.93	13

SD: standard deviation. *Data are represented as median (range).

**Table 5.** Quality score calculated using the Methodological Index for Non-Randomized Studies.

Study	MINORS item												Total
	1)	2)	3)	4)	5)	6)	7)	8)	9)	10)	11)	12)	
Kim et al., 2012	2	2	1	2	0	2	2	1	2	2	1	1	18
Lin et al., 2014	2	1	1	2	0	1	2	1	2	2	2	1	17
Hsiao et al., 2015	2	2	1	2	0	2	2	1	2	2	2	1	19
Takahashi et al., 2015	2	2	1	2	0	2	1	1	2	2	1	1	17
Piessen et al., 2015	2	1	1	2	0	2	2	1	2	2	2	1	18
Xue et al., 2015	2	2	1	2	0	2	2	1	2	2	0	1	17
Khoo et al., 2017	2	2	1	2	0	2	2	1	2	1	1	1	17
Qiu et al., 2017	2	1	1	2	0	2	2	1	2	2	2	1	18
Lian et al., 2017	2	1	1	2	0	2	2	1	2	2	2	1	18

0: not reported; 1: reported but inadequate; 2: reported and adequate. 1) a clearly stated aim; 2) inclusion of consecutive patients; 3) prospective collection of data; 4) end points appropriate to the aim of the study; 5) unbiased assessment of study end point; 6) follow-up period appropriate to the aim of the study; 7) loss of follow-up less than 5%; 8) prospective calculation of the study size; 9) an adequate control group; 10) contemporary groups; 11) baseline equivalence groups; 12) adequate statistical analyses.

### Meta-Analyses

Initially, in addition to the selected outcome measures, we also conducted a statistical analysis regarding the size of tumors submitted to intervention by LAP and OS, with data being obtained from seven studies^12,16-18,20,23,27^ (Table 6).

**Table 6.** Tumor size.

Study or Subgroup	Laparoscopic Surgery			Open Surgery				Mean Difference	
	Mean (cm)	SD (cm)	Total	Mean (cm)	SD (cm)	Total	Weight (%)	IV, Fixed, 95%CI	Year
Kim et al. 2012	6.1	1.3	24	7.2	1.7	14	12.6	- 1.10 [-2.13, -0.07]	2012
Hsiao et al. 2015	6.3	1.1	18	6	0.9	21	32.8	0.30 [-0.34, 0.94]	2014
Lin et al. 2014	7.2	1.6	23	7.3	1.5	23	16.6	-0.10 [-1.00, 0.80]	2014
Takahashi et al. 2015	6.025	0.79	12	8.325	5.17	15	1.9	-2.30 [-4.95, 0.35]	2015
Khoo et al. 2017	7.04	1.5	23	7.53	1.875	36	17.8	-0.49 [-1.36, 0.38]	2016
Qiu et al. 2017	7	1.4	24	7.6	1.75	24	16.6	-0.60 [-1.50, 0.30]	2017
Lian et al. 2017	6.5	3.33	11	6.5	3.33	11	1.7	0.00 [-2.78, 2.78]	2017
**Total (95%CI)**			**135**			**144**	**100.0**	**-0.29 [-0.65, 0.08]**	

Heterogeneity: ChI^2^=8.74, df=6 (p=0.19); I^2^=31%. Test for overall effect: Z=1.54 (p=0.12). SD: standard deviation. CI: confidence interval.

No statistically significant differences were observed between the two groups (MD=−0.29; 95%CI −0.65; 0.08; p=0.12). Between groups, the heterogeneity was not substantial (I^2^=31%).

### Intraoperative Outcomes (Operative Time and Intraoperative Blood Loss)

Operative time (with a sample of 156 patients from LAP approach and 208 patients from OS) and intraoperative blood loss (with a sample of 132 patients from LAP approach and 194 from OS) were reported in eight^12,16-18,20,23,27,28^ and seven^12,16,18,20,23,27,28^ studies (Table 7-A and 7-B respectively). No statistically significant differences were found in operative time (MD=18.90; 95%CI −13.19; 51.00; p=0.25) and heterogeneity between studies was substantial (I^2^=89%). However, the LAP approach is associated with statistically lower amounts of intraoperative blood loss (MD=−30.82; 95%CI −54.93; −6.71; p=0.01). The heterogeneity between studies was substantial (I^2^=59%).

**Table 7.** Intraoperative outcomes: (A) operative time; (B) intraoperative blood loss.

(A)									
Study or Subgroup	Laparoscopic Surgery			Open Surgery				Mean Difference	
	Mean (min)	SD (min)	Total	Mean (min)	SD (min)	Total	Weight(%)	IV, Random, 95%CI	Year
Kim et al. 2012	119.8	62.2	24	154.3	53.5	14	12.0	-34.50 [-71.98, 2.98]	2012
Hsiao et al. 2015	146.6	50.2	18	113.3	42.9	21	12.8	33.30 [3.73, 62.87]	2014
Lin et al. 2014	124.1	50.3	23	196.5	64.8	23	12.4	-72.40 [-105.92, -38.88]	2014
Takahashi et al. 2015	123.75	44.75	12	119.5	44.22	15	12.4	4.25 [-29.54, 38.04]	2015
Xue et al. 2015	128.9	38.2	19	106	39.2	62	13.6	22.90 [3.15, 42.65]	2015
Khoo et al. 2017	210	81.25	23	105	48.75	36	12.1	105.00 [68.17, 141.83]	2016
Lian et al. 2017	197.46	59.774	13	129.23	56	13	11.2	68.23 [23.71, 112.75]	2017
Qiu et al. 2017	131	44	24	103	30	24	13.5	28.00 [6.69, 49.31]	2017
**Total (95%CI)**			**156**			**208**	**100.0**	**18.90 [-13.19, 51.00]**	
Heterogeneity: Tau^2^=1868.63; ChI^2^=64.49, df=7 (p<0.00001); I^2^=89%.									
Test for overall effect: Z=1.15 (p=0.25).									
SD: standard deviation. CI: confidence interval.									

(B)									
Study or Subgroup	Laparoscopic Surgery			Open Surgery				Mean Difference	
	Mean (min)	SD (min)	Total	Mean (min)	SD (min)	Total	Weight(%)	IV, Random, 95%CI	Year
Hsiao et al. 2015	42.2	40.7	18	51.4	58.4	21	20.0	-9.20 [-40.46, 22-06]	2014
Lin et al. 2014	35.6	28.3	23	127.8	116.8	23	13.4	-92.20 [-141.32, -43.08]	2014
Takahashi et al. 2015	202.5	244.65	12	255	262.69	15	1.5	-52.50 [-244.42, 139.42]	2015
Xue et al. 2015	34.1	26.5	19	60.4	60.4	62	25.3	-26.30 [-45.48, -7.12]	2015
Khoo et al. 2017	78.26	75	23	125	128.47	36	12.8	-50.21 [-101.27, 0.85]	2016
Qiu et al. 2017	73	36	24	51	105	24	22.7	-32.00 [-56.98, -7.02]	2017
Lian et al. 2017	207.5	175.86	13	86.84	105	13	4.4	102.50 [-4.12, 209.12]	2017
**Total (95%CI)**			**132**			**194**	**100.0**	**-30.82 [-54.93, -6.71]**	
Heterogeneity: Tau^2^=503.33; ChI^2^=14.60, df=6 (p=0.02); I^2^=59%.									
Test for overall effect: Z=2.51 (p=0.01).									
SD: standard deviation. CI: confidence interval.									

### Short-Term Postoperative Outcomes (Time to Oral Intake, Time to first Flatus, and Length of Hospital Stay) and Postoperative Complications

Data on time to oral intake were reported in four studies^16,18,20,23^ (Table 8-A) and data on time to first flatus in four studies^18,20,23,28^ as well (Table 8-B). The LAP approach required a statistically significantly shorter time to oral feeding (MD=−0.54; 95%CI −0,84; −0,24); p<0.01), with an inter-study heterogeneity not substantial (I^2^=30%). Also, in terms of time to first flatus, the LAP route showed statistically lower values (MD=−0.45; 95%CI −0.72; −0.18; p<0.01). The heterogeneity between studies was I^2^=39%.

Eight studies^12,16-18,20,23,27,28^ reported the length of hospital stay, with 156 patients in the LAP approach group and 208 in the OS group (Table 8-C). A substantial inter-study heterogeneity was found (I^2^=70%). The LAP approach is associated with a statistically significantly shorter length of hospital stay than the OS approach (MD=−1.83; 95%CI −3.12; −0.53; p=0.01).

Seven studies^16-18,20,22,23,27^ reported data regarding the occurrence of postoperative complications, with a sample of 209 patients in the LAP group and 218 in the OS group (Table 8-D). No statistically significant differences were found (RR=0.63; 95%CI 0.38; 1.05; p=0.08), and no heterogeneity between studies was obtained (I^2^=0%).

**Table 8.** Short-term postoperative outcomes: (A) time to oral intake; (B) time to first flatus; (C) length of hospital stay and (D) postoperative complications.

(A)									
Study or Subgroup	Laparoscopic Surgery			Open Surgery				Mean Difference	
	Mean (days)	SD (days)	Total	Mean (days)	SD (days)	Total	Weight(%)	IV, Fixed, 95%CI	Year
Lin et al. 2014	2.3	1.5	23	3.5	2.3	23	7.0	-1.20 [-2.32, -0.08]	2014
Khoo et al. 2017	2.25	0.75	23	3	1.25	36	34.0	-0.75 [-1.26, -0.24]	2016
Qiu et al. 2017	3.2	0.6	24	3.6	0.8	24	55.3	-0.40 [-0.80, 0.00]	2017
Lian et al. 2017	4.77	1.48	13	4.23	2.45	13	3.7	0.54 [-1.02, 2.10]	2017
**Total (95%CI)**			**83**			**96**	**100.0**	**-0.54 [-0.84, -0.24]**	
Heterogeneity: ChI^2^=4.30, df=3 (p=0.23); I^2^=30%.									
Test for overall effect: Z=3.56 (p=0.0004).									

(B)									
Study or Subgroup	Laparoscopic Surgery			Open Surgery				Mean Difference	
	Mean (days)	SD (days)	Total	Mean (days)	SD (days)	Total	Weight (%)	IV, Fixed, 95%CI	Year
Lin et al. 2014	1.9	1.2	23	3	1	23	17.4	-1.10 [-1.74, -0.46]	2014
Xue et al. 2015	3.5	1.4	19	3.9	1	62	15.5	-0.40 [-1.08, 0.28]	2015
Qiu et al. 2017	2.2	0.5	24	2.5	0.7	24	59.9	-0.30 [-0.64, 0.04]	2017
Lian et al. 2017	3.69	0.75	13	3.92	1.66	13	7.2	-0.23 [-1.22, 0.76]	2017
**Total (95%CI)**			**79**			**122**	**100.0**	**-0.45 [-0.72, -0.18]**	
Heterogeneity: ChI^2^=4.92, df=3 (p=0.18); I^2^=39%.									
Test for overall effect: Z=3.31 (p=0.0009).									

(C)									
Study or Subgroup	Laparoscopic Surgery			Open Surgery				Mean Difference	
	Mean (days)	SD (days)	Total	Mean (days)	SD (days)	Total	Weight (%)	IV, Random, 95%CI	Year
Kim et al. 2012	4.8	1.8	24	9.2	3.2	14	14.6	-4.40 [-6.22, -2.58]	2012
Hsiao et al. 2015	8.4	2.9	18	9.6	2.4	21	15.5	-1.20 [-2.80, 0.49]	2014
Lin et al.2014	7.2	1.6	23	10.1	2.6	23	17.6	-2.90 [-4.15, -1.65]	2014
Takahashi et al. 2015	14.25	7.46	12	18.25	8.01	15	4.0	-4.00 [-9.85, 1.85]	2015
Xue et al. 2015	7.2	3.8	19	9	4.8	62	13.6	-1.80 [-3.88, 0.28]	2015
Khoo et al. 2017	21.22	17.5	23	21.12	15	36	2.0	0.10 [-8.57, 8.77]	2016
Lian et al. 2017	7.92	2.66	13	6.69	1.93	13	15.0	1.23 [-0.56, 3.02]	2017
Qiu et al. 2017	6.6	2.2	24	8.1	2.3	24	17.5	-1.50 [-2.77, -0.23]	2017
**Total (95%CI)**			**156**			**208**	**100.0**	**-1.83 [ [-3.12, -0.53]**	
Heterogeneity: Tau^2^=2.08; ChI^2^=23.21, df=7 (p=0.002); I^2^=70%.									
Test for overall effect: Z=2.77 (p=0.006).									

(D)							
Study or Subgroup	Laparoscopic Surgery		Open Surgery			Risk Ratio	
	Events	Total	Events	Total	Weight (%)	IV, Fixed, 95%CI	Year
Kim et al. 2012	1	24	0	14	2.6	1.80 [0.08, 41.42]	2012
Lin et al. 2014	2	23	3	23	8.9	0.67 [0.12, 3.62]	2014
Piessen et al. 2015	11	90	21	93	56.9	0.54 [0.28, 1.06]	2015
Takahashi et al. 2015	1	12	1	15	3.6	1.25 [0.09, 17.98]	2015
Khoo et al. 2017	2	23	5	36	10.6	0.63 [0.13, 2.96]	2016
Qiu et al. 2017	3	24	5	24	14.8	0.60 [0.16, 2.23]	2017
Lian et al. 2017	1	13	0	13	2.6	3.00 [0.13, 67.51]	2017
**Total (95%CI)**		**209**			**218**	**100.0**	**0.63 [0.38, 1.05]**	
Heterogeneity: ChI^2^=1.86, df=6 (p=0.93); I^2^=0%.							
Test for overall effect: Z=1.78 (p=0.08).							
SD: standard deviation. CI: confidence interval.								

### Short- and Long-Term Oncological Outcomes: R0 Resection and Recurrence Rate

Eight studies^12,16-18,20,22,23,27^ reported data regarding locoregional disease recurrence (Table 9-A), with 227 patients in the LAP approach group and 239 in the OS group. The recurrence rate for patients undergoing LAP was 5.29% (n=12/227), compared to a recurrence rate of 9.8% (n=23/239) in patients undergoing OS. The summary analytical measure was not statistically significant (RR=0.57; 95%CI 0.29; 1.09; p=0.09). Inter-study heterogeneity was null (I^2^=0%).

**Table 9.** Oncological outcomes: (A) recurrence rate; (B) R0 resection.

(A)							
Study or Subgroup	Laparoscopic Surgery		Open Surgery			Risk Ratio	
	Events	Total	Events	Total	Weight (%)	IV, Fixed, 95%CI	Year
Kim et al. 2012	1	24	4	14	9.9	0.15 [0.02, 1.18]	2012
Hsiao et al. 2015	1	18	0	21	4.4	3.47 [0.15, 80.35]	2014
Lin et al. 2014	3	23	5	23	25.2	0.60 [0.16, 2.22]	2014
Piessen et al. 2015	5	90	7	93	35.0	0.74 [0.24, 2.24]	2015
Takahashi et al. 2015	1	12	2	15	8.3	0.63 [0.06, 6.09]	2015
Khoo et al. 2017	0	23	2	36	4.8	0.31 [0.02, 6.15]	2016
Qiu et al. 2017	1	24	2	24	7.9	0.50 [0.05, 5.15]	2017
Lian et al. 2017	0	13	1	13	4.5	0.33 [0.01, 7.50]	2017
**Total (95%CI)**		**227**		**239**	**100.0**	**0.57 [ 0.29, 1.09]**	
Heterogeneity: ChI^2^=3.41, df=7 (p=0.84); I^2^=0%.							
Test for overall effect: Z=1.70 (p=0.09).							

(B)							
Study or Subgroup	Laparoscopic Surgery		Open Surgery			Risk Ratio	
	Events	Total	Events	Total	Weight (%)	M-H, Fixed, 95%CI	Year
Kim et al. 2012	24	24	14	14	25.1	1.00 [0.90, 1.12]	2012
Hsiao et al. 2015	17	18	19	21	24.3	1.04 [0.87, 1,25]	2014
Qiu et al. 2017	23	24	23	24	31.9	1.00 [0.89, 1.13]	2017
Lian et al. 2017	13	13	13	13	18.7	1.00 [0.87, 1.15]	2017
**Total (95%CI)**		**79**		**72**	**100.0**	**1.01 [0.94, 1.08]**	
Heterogeneity: ChI^2^=0.21, df=3 (p=0.98); I^2^=0%.							
Test for overall effect: Z=0.30 (p=0.76).							
SD: standard deviation. CI: confidence interval.							

Table 9-B shows that four studies^12,17,18,23^ reported data regarding the possibility of tumor R0 resection. The results showed an absence of heterogeneity (I^2^=0%), and no statistically significant differences were detected regarding the two different approaches (p=0.76).

### Subgroup Analysis

We conducted a subgroup analysis in order to explore the heterogeneity obtained regarding the results of operative time, intraoperative blood loss, and length of hospital stay.

When considering operative time, the test for subgroup differences indicates that there were no statistically significant differences (p=0.20; I^2^=39.2%), suggesting that the inequality between methodological approaches used by the two subgroups of studies is unlikely to explain the high heterogeneity (Table 10-A). Table 10-B and 10-C shows the results of subgroup analyses regarding intraoperative blood loss and length of hospital stay. In both continuous analyses, heterogeneity was eliminated for the subgroup referent to studies that only included wedge tumor resections, and no statistically significant differences were found between the two approaches (intraoperative blood loss: I^2^=0%, p=0.39; length of hospital stay: I^2^=0%, p=0.63). However, no statistically significant differences were found between the subgroups in both blood loss (p=0.64) and length of hospital stay (p=0.66).

**Table 10.** Subgroup analysis: (A): operative time; (B) intraoperative blood loss; (C) length of hospital stay.

(A)									
Study or Subgroup	Laparoscopic Surgery			Open Surgery				Mean Difference	
	Mean (min)	SD (min)	Total	Mean (min)	SD (min)	Total	Weight(%)	IV, Random, 95%CI	Year
2.11 Wedge Resection									
Hsiao et al. 2015	146.6	50.2	18	113.3	42.9	21	12.8	33.30 [3.73, 62.87]	2014
Takahashi et al. 2015	123.75	44.75	12	119.5	44.22	15	12.4	4.25 [-29.54, 38.04]	2015
Khoo et al. 2017	210	81.25	23	105	48.75	36	12.1	105.00 [68.17, 141.83]	2016
**Subtotal (95%CI)**			**53**			**72**	**37.2**	**46.93 [-8.03, 101.90]**	
Heterogeneity: Tau^2^=2067.81; ChI^2^=16.35; df=2 (p=0.0003); I^2^=88%.									
Test for overall effect: Z=1.67 (p=0.09).									
All Surgeries									
Kim et al. 2012	119.8	62.2	24	154.3	53.5	14	12.0	-34.50 [-71.98, 2.98]	2012
Lin et al. 2014	124.1	50.3	13	196.5	64.8	23	12.4	-72.40 [-105.92, -38.88]	2014
Xue et al. 2015	128.9	38.2	19	106	39.2	62	13.6	22.90 [3.15, 42.65]	2015
Qiu et al. 2017	131	44	24	103	30	24	13.5	28.00 [6.69, 49.31]	2017
Lian et al. 2017	197.46	59.774	23	129.23	56	13	11.2	68.23 [23.71, 112.75]	2017
**Subtotal (95%CI)**			**103**			**136**	**62.8**	**2.30 [-38.11, 42.71]**	
Heterogeneity: Tau^2^=1857.86; ChI^2^=39.57, df=4 (p<0.00001); I^2^=90%.									
Test for overall effect: Z=0.11 (p=0.91).									
**Total (95%CI)**			**156**			**208**	**100.0**	**18.90 [-13.19, 51.99]**	
Heterogeneity: Tau^2^=1868.63; ChI^2^=64.49, df=7 (p<0.00001); I^2^=89%.									
Test for overall effect: Z=1.15 (p=0.25).									
Test for subgroup differences: ChI^2^=1.64, df=1 (p=0.20); I^2^=39.2%.									

(B)									
Study or Subgroup	Laparoscopic Surgery			Open Surgery				Mean Difference	
	Mean (mL)	SD (mL)	Total	Mean (mL)	SD (mL)	Total	Weight (%)	IV, Random, 95%CI	Year
2.11 Wedge Resection									
Hsiao et al. 2015	42.2	40.7	18	51.4	58.4	21	20.0	-9.20 [-40.46, 22.06]	2014
Takahashi et al. 2015	202.5	244.65	12	255	262.69	15	1.5	-52.50 [-244.42, 139.42	2015
Khoo et al. 2017	78.26	75	23	128.47	125	36	12.8	-50.21 [-101.27, 0.85]	2016
**Subtotal (95%CI)**			**53**			**72**	**3**4.3	**-20.99 [-47.40, 5.42]**	
Heterogeneity: Tau^2^=0.00; ChI^2^=1.91; df=2 (p=0.39); I^2^=0%.									
Test for overall effect: Z=1.56 (p=0.12).									
All Surgeries									
Lin et al. 2014	35.6	28.3	23	127.8	116.8	23	13.4	-92.20 [-141.32, -43.08]	2014
Xue et al. 2015	34.1	26.5	19	60.4	60.4	62	25.3	-26.30 [-45.48, -7.12]	2015
Qiu et al. 2017	73	36	24	105	51	24	22.7	-32.00 [-56.98, -7.02]	2017
Lian et al. 2017	207.5	175.86	13	105	86.84	13	4.4	102.50 [-4.12, 209.12]	2017
**Subtotal (95%CI)**			**79**			**122**	**65.7**	**-31.66 [-68.04, 4.71]**	
Heterogeneity: Tau^2^=881.71; ChI^2^=12.22, df=3 (p=0.007); I^2^=75%.									
Test for overall effect: Z=1.71 (p=0.09).									
**Total (95%CI)**			**132**			**194**	**100.0**	**-30.82 [-54.93, -6.71]**	
Heterogeneity: Tau^2^=503.33; ChI^2^=14.60, df=6 (p=0.02); I^2^=59%.									
Test for overall effect: Z=2.51 (p=0.01).									
Test for subgroup differences: ChI^2^=0.22, df=1 (p=0.64); I^2^=0%.									

(C)									
Study or Subgroup	Laparoscopic Surgery			Open Surgery				Mean Difference	
	Mean (days)	SD (days)	Total	Mean (days)	SD (days)	Total	Weight (%)	IV, Random, 95%CI	Year
2.11 Wedge Resection									
Hsiao et al. 2015	8.4	2.9	18	9.6	2.4	21	15.5	-1.20 [-2.89, 0.49]	2014
Takahashi et al. 2015	14.25	7.46	12	18.25	8.01	15	4.0	-4.00 [-9.85, 1.85]	2015
Khoo et al. 2017	21.22	17.5	23	21.12	15	36	2.0	0.10 [-8.57, 8.77]	2016
**Subtotal (95%CI)**			**53**			**72**	**21.5**	**-1.36 [-2.96, 0.23]**	
Heterogeneity: Tau^2^=0.00; ChI^2^=0.93; df=2 (p=0.63); I^2^=0%.									
Test for overall effect: Z=1.68 (p=0.09).									
**All Surgeries**									
Kim et al. 2012	4.8	1.8	24	9.2	3.2	14	14.8	-4.40 [-6.22, -2.58]	2012
Lin et al. 2014	7.2	1.6	13	10.1	2.6	23	17.6	-2.90 [-4.15, -1.65]	2014
Xue et al. 2015	7.2	3.8	19	9	4.8	62	13.6	-1.80 [-3.88, 0.28]	2015
Lian et al. 2017	7.92	2.66	13	6.69	1.93	13	15.0	1.23 [-0.56, 3.02]	2017
Qiu et al. 2017	6.6	2.2	24	8.1	2.3	24	17.5	-1.50 [-2.77, -0.23]	2017
**Subtotal (95%CI)**			**103**			**136**	**78.5**	**-1.89 [-3.55, -0.23]**	
Heterogeneity: Tau^2^=2.88; ChI^2^=21.82, df=7 (p=0.0002); I^2^=82%.									
Test for overall effect: Z=2.23 (p=0.03).									
**Total (95%CI)**			**156**			**208**	**100.0**	**-1.83 [-3.12, -0.53]**	
Heterogeneity: Tau^2^=2.08; ChI^2^=23.21, df=7 (p=0.002); I^2^=70%.									
Test for overall effect: Z=2.77 (p=0.006).									
Test for subgroup differences: ChI^2^=0.20, df=1 (p=0.66); I^2^=0%.									
SD: standard deviation. CI: confidence interval.									

## DISCUSSION

GISTs are mesenchymal tumors that arise in the wall of the GI tract, and, due to the increase in upper GI endoscopy, the detection of these tumors in the stomach has suffered a significant increase, becoming the location where they are most frequently detected^20,22^. One of the most relevant prognostic factors indicating aggressive behavior of a GIST is its size^9^. As surgical intervention is the main form of treatment, it is important, through endoscopic techniques and imaging methods, to evaluate the size of the tumor, its location, and possible local invasion or concomitant metastasis, prior to its resection^20,27^. A statistical analysis of the size of the tumors was performed and no statistically significant differences were observed between the two compared approaches in the selected articles.

With the clear advantages of minimally invasive surgery (less pain, smaller incisions, shorter time to recovery of bowel function, and shorter hospital stay), the laparoscopic approach is often the preferred choice for many surgeons^23^. However, the NCCN guidelines^8^ only present clear recommendations regarding the use of the laparoscopic approach in tumors smaller than 5 cm, while the ESMO clinical guidelines^6^ even discourage the use of this technique in large tumors. Some concerns arise when deciding to use laparoscopic approach for the treatment of larger GISTs: the necessity to prevent tumor rupture during tumor management (which is associated a higher risk of recurrence), avoiding subsequent peritoneal implantation, and the difficulty the surgeon faces when extracting the surgical specimen through small incisions^12^.

### Intraoperative Outcomes

In our meta-analysis, there were no statistically significant differences in operative time. This may be due to the need of performing larger incisions in order to allow the removal of bigger tumors when using the laparoscopic approach. In addition, it is also likely that the increasing expertise of surgeons in this technique and the use of progressively more sophisticated instruments contribute to the decrease in time^23,27^. As for intraoperative blood loss, the laparoscopic approach showed statistically significant lower values, which may be due to the fact that LAP is performed using a built-in camera that provides surgeons a more detailed visual field, thus allowing greater precision during the operative and avoiding the inappropriate handling of small vessels and other anatomical structures. Simultaneously, smaller incision sizes may also justify the lower losses with laparoscopy^4,23^.

### Short-Term Postoperative Outcomes and Postoperative Complications

With regard to short-term postoperative outcomes, time to first flatus, time to oral intake, and length of hospital stay, all were shown to occur earlier in the laparoscopic approach, with this difference being statistically significant. These results are in conformity with the inherent advantages of this type of approach^2^. Smaller incision sizes allow patients to have less postoperative pain and earlier mobilization. In addition, with less handling of the GI tract during surgery, patients recover bowel function sooner, allowing an earlier return to oral intake and, ultimately, a shorter hospital stay^4,23^. Regarding the number of postoperative complications, no significant differences were detected between the two approaches. These data support that, due to its reduced invasiveness, in terms of safety and feasibility, laparoscopy seems to be an option.

### Short- and Long-Term Oncological Outcomes

One of the important points when considering the use of a new surgical approach is that it demonstrates oncological results that are not inferior when compared to the gold standard method, proving its noninferiority. The goal of surgical treatment of GISTs is to achieve resection with free margins^1^ and, when comparing the two types of surgery approaches, no statistically significant differences were observed. As already stated, tumor rupture should be avoided^5^. If this happens, it is associated with higher recurrence rates. Our results showed no differences in recurrence rates, which may be associated with the high level of experience that surgeons are acquiring in this approach. Furthermore, the removal of the surgical piece using a protective plastic bag provides a decreased risk of recurrence at the trocar entry ports^8^.

### Subgroup Analysis

In order to try to explore the high heterogeneity obtained in the analysis of some outcomes (intraoperative time, intraoperative blood loss, and length of hospital stay), a subgroup analysis was performed, in which studies that only considered wedge resections were separated from those that included several types of surgery. Since wedge resection is a methodologically simpler approach, one could expect that it would lead to shorter operative and hospitalization times, thereby explaining the variability obtained among the various studies. However, this did not happen, and although heterogeneity in some outcomes (intraoperative blood loss and length of hospital stay) was eliminated in the wedge resection subgroup, this analysis was not statistically significant in terms of differences between the two subgroups evaluated. We were thus unable to explain the high heterogeneity obtained, and the essence of the problem may be due to the lack of data regarding methodological diversity or due to the presence of differences in outcome assessment, given the still limited experience with the laparoscopic approach in large tumors.

### Study Limitations and Future Perspectives

Laparoscopy, in addition to the clear advantages of being a minimally invasive approach with low incidence of postoperative complications, has proven to have similar oncological results, shorter times to oral intake, first flatus, and hospitalization. Huang et al.^13^ have described similar long-term outcomes to OS when performed on gastric GISTs in unfavorable sites, so the decision to pursue a laparoscopic approach should always depend on the experience of the surgeon.

This review has some limitations, so its interpretation should be made with caution. Regarding the retrospective cohort studies included, there is always some risk of selection bias, due to the lack of randomization, which may lead to the treatment effects being higher than the reality. Even though the studies tried to control possible confounders by presenting similar baseline characteristics in the different types of approach, the truth is, it is practically never possible to assume that all factors that can affect prognosis and response to a treatment are known. Also, the lack of blinding, observed in all our studies, in the evaluation of outcomes may lead to an overestimation of the results obtained. However, this situation is more relevant in subjective outcomes, so our analysis should not be so affected. Adding to this, the studies included in our meta-analysis comprised treatments performed over long periods of time, which, due surgeon’s increasing experience, technological developments, and changes in hospital practices, may have affected the results. Finally, the high heterogeneity obtained in some outcomes, which could not be explained by the subgroup analysis performed, should also be taken into consideration.

These results are encouraging for the development of further studies, ideally prospective and randomized, that validate the role of laparoscopy in the treatment of gastric GISTs larger than 5 cm. If this is established as the standard of treatment in experienced centers, the benefits of laparoscopy could be more widely offered to patients with this pathology.

## CONCLUSIONS

The laparoscopic approach in GISTs larger than 5 cm compared to the OS approach is a technically safe and feasible surgical method with similar oncological results, so its application may become the standard in the future.
